# Geographic information systems for occupational cancer surveillance: a scoping review

**DOI:** 10.15649/cuidarte.4747

**Published:** 2025-10-03

**Authors:** Diana Carolina Sanchez, Lorena Lisbeth Talero, Jose Ferney Mejia-Duarte, Alejandra Mendoza-Monsalve, Maricel Licht-Ardila, Alexandra Hurtado-Ortiz

**Affiliations:** 1 Universidad El Bosque, Bogotá, Colombia. E-mail: sanchezdiana@unbosque.edu.co Universidad El Bosque Bogotá Colombia sanchezdiana@unbosque.edu.co; 2 Universidad El Bosque, Bogotá, Colombia. E-mail: ltalerof@unbosque.edu.co Universidad El Bosque Bogotá Colombia ltalerof@unbosque.edu.co; 3 Universidad El Bosque, Bogotá, Colombia. E-mail: jfmejiad@unbosque.edu.co Universidad El Bosque Bogotá Colombia jfmejiad@unbosque.edu.co; 4 Fundación Cardiovascular de Colombia. Piedecuesta, Santander, Colombia. E-mail: marymendozamonsalve@fcv.org Fundación Cardiovascular de Colombia Santander Colombia marymendozamonsalve@fcv.org; 5 Fundación Cardiovascular de Colombia. Piedecuesta, Santander, Colombia. E-mail: maricellicht@fcv.org Fundación Cardiovascular de Colombia Santander Colombia maricellicht@fcv.org; 6 Fundación Cardiovascular de Colombia. Piedecuesta, Santander, Colombia. E-mail: alexandrajhop@gmail.com Fundación Cardiovascular de Colombia Santander Colombia alexandrajhop@gmail.com

**Keywords:** Geographic Information Systems, Occupational Medicine, Epidemiologic Surveillance, Neoplasms, Environmental Exposure, Spatial Analysis, Sistemas de Información Geográfica, Medicina Ocupacional, Vigilancia Epidemiológica, Neoplasias, Exposición Ambiental, Análisis Espacial, Sistemas de Informação Geográfica, Medicina Ocupacional, Vigilância Epidemiológica, Neoplasias, Exposição Ambiental, Análise Espacial

## Abstract

**Introduction:**

Geographic Information Systems (GIS) are key tools for managing spatial data and understanding the determinants of occupational cancer.

**Objective:**

To evaluate the applications, advantages, and limitations of GIS in the surveillance of occupational cancer.

**Materials and Methods:**

: A systematic scoping review was conducted using PubMed, Embase, Scopus, and Bireme databases, following the Population, Context, and Concept (PCC) framework outlined in the Joanna Briggs Institute (JBI) methodological guidelines and the PRISMA ScR. A semi-automated process supported by Rayyan® software was employed for study selection. The variables identified were transferred to a spreadsheet for qualitative analysis and synthesis.

**Results:**

A total of 55 articles were included, addressing various cancer types and exposure to industrial emissions and potentially carcinogenic pollutants. The most commonly used GIS, spatial analysis methodologies, and the main advantages and limitations of their use were identified in monitoring morbidity and mortality, equity, timeliness, coverage, and access to health services, as well as in modeling environmental agents.

**Discussion:**

GIS advance cancer research by integrating and analyzing diverse datasets, mapping cases, and identifying risk factors. Challenges include data accuracy, incomplete records, and omission of socioeconomic variables. Despite limitations, GIS support cancer surveillance, occupational health policies, and prevention plans.

**Conclusion:**

GIS are valuable tools for cancer surveillance, as they improve understanding of the geographic patterns of exposure and associated variables, providing critical insights for public policy formulation, healthcare planning, and preventive strategies.

## Resumen

**Introducción:**

Los Sistemas de Información Geográfica (SIG) son herramientas informáticas diseñadas para evaluar datos espaciales y constituyen un instrumento crucial para comprender la enfermedad y sus determinantes.

**Objetivo:**

Realizar una revisión sistemática del alcance para evaluar las aplicaciones, ventajas y limitaciones de los SIG en la vigilancia del cáncer ocupacional.

**Materiales y métodos:**

Se llevó a cabo una búsqueda sistemática exploratoria en las bases de datos PubMed, Embase, Scopus y Bireme, siguiendo el marco de Población, Contexto y Concepto (PCC) de las directrices metodológicas del Instituto Joanna Briggs (JBI) y el protocolo PRISMA-ScR. Para la selección de estudios se utilizó un proceso semiautomatizado con el software Rayyan®. Las variables identificadas fueron transferidas para su posterior análisis cualitativo. Se incluyeron 55 artículos que cumplieron con los criterios de inclusión.

**Resultados:**

La revisión abarcó estudios sobre diversos tipos de cáncer, emisiones industriales y contaminantes carcinógenos relevantes para la salud ocupacional, destacando las herramientas SIG más utilizadas, las metodologías de análisis espacial y las principales ventajas y limitaciones en la vigilancia del cáncer. Los estudios reportaron indicadores de morbilidad y mortalidad, equidad, oportunidad, cobertura en salud y acceso, así como modelos de exposición a agentes ambientales.

**Discusión:**

Los SIG fortalecen significativamente la investigación en cáncer al integrar y analizar diversos conjuntos de datos, mapear casos e identificar factores de riesgo. Entre los desafíos se encuentran la precisión de los datos, la existencia de registros incompletos y la omisión de variables socioeconómicas. A pesar de estas limitaciones, los SIG apoyan la vigilancia del cáncer, las políticas de salud ocupacional y los planes de prevención, al visibilizar riesgos de exposición, desigualdades en salud y oportunidades de mejora.

**Conclusiones:**

Los SIG son herramientas cada vez más valiosas en la vigilancia del cáncer, ya que permiten comprender mejor los patrones geográficos de exposición y las variables asociadas, ofreciendo insumos clave para la formulación de políticas públicas, la planificación en salud y las estrategias preventivas.

## Resumo

**Introdução:**

Sistemas de Informação Geográfica (SIG) são ferramentas computadorizadas projetadas para avaliar dados espaciais e são instrumentos cruciais para a compreensão da doença e seus determinantes.

**Objetivo:**

Realizar uma revisão sistemática de escopo para avaliar as aplicações, vantagens e limitações dos SIG na vigilância do câncer de relevância ocupacional.

**Materiais e métodos:**

Uma busca sistemática de escopo foi realizada utilizando PubMed, Embase, Scopus e Bireme, aderindo à estrutura População, Contexto e Conceito (PCC) descrita nas diretrizes metodológicas do Instituto Joanna Briggs (JBI) e o protocolo PRISMA ScR. Um processo semiautomatizado com o software Rayyan® foi empregado para a seleção dos estudos. As variáveis identificadas foram transferidas para análise subsequente, para conduzir uma pesquisa qualitativa. 55 artigos atenderam aos critérios de inclusão.

**Resultados:**

A revisão abrangeu estudos sobre vários tipos de câncer, emissões industriais e poluentes carcinogênicos relevantes para a saúde ocupacional; destacando ferramentas de SIG comumente utilizadas, metodologias de análise espacial e as vantagens e limitações dos SIG no monitoramento do câncer. Os estudos relataram indicadores de morbidade e mortalidade, equidade, pontualidade, cobertura e acesso à saúde, juntamente com indicadores de modelagem de agentes ambientais.

**Discussão:**

O SIG avança significativamente a pesquisa sobre câncer ao integrar e analisar diversos conjuntos de dados, mapear casos e identificar fatores de risco. Os desafios incluem a precisão dos dados, registros incompletos e variáveis socioeconômicas negligenciadas. Apesar das limitações, o SIG apoia a vigilância do câncer, as políticas de saúde ocupacional e os planos de prevenção, destacando os riscos de exposição, as disparidades na assistência à saúde e as oportunidades para melhorias futuras.

**Conclusões:**

O SIG é uma ferramenta cada vez mais valiosa na vigilância do câncer, aprimorando a compreensão dos padrões geográficos de exposição e variáveis associadas, fornecendo insights críticos para políticas públicas, planejamento de saúde e estratégias preventivas.

## Introduction

Geographic Information Systems (GIS) are computerized tools capable of integrating, assembling, storing, and manipulating spatial or cartographic data to reveal the actual conditions of a georeferenced variable under study^1^. In the health field, GIS have been employed by combining demographic, environmental, and social variables to enable georeferencing. This approach has promoted the creation of health event maps that facilitate the identification of risk factors, the distribution of services, and the availability of resources (physical, human, and infrastructural, among others). These maps also highlight specific social determinants of health, supporting interventions for damage control or the reorganization of health services based on population needs, a method grounded in the principles of nosogeography^1^,^2^. 

In oncology, evidence of mortality mapping dates back to the 1800s in England, where associations between cancer and environmental exposure were first established. Today, GIS applications in oncology surveillance are reflected in initiatives such as those led by the American Cancer Society, the International Agency for Research on Cancer (IARC), and the Union for International Cancer Control^3^. Additionally, noteworthy local developments in countries like Spain^4^, India^5^, Argentina^6^, Chile^7^, and Colombia^8^ have demonstrated the usefulness of GIS in cancer studies. 

Cancer remains a major global health concern and the leading cause of death worldwide. According to the World Health Organization (WHO), it accounted for 10 million deaths in 2020^9^. Furthermore, cancer cases are projected to increase by 32% by 2030, with more than 5 million new diagnoses annually in the Americas, driven by demographic shifts^10^. Many cancer factors overlap with those of other non-communicable diseases, such as tobacco use, harmful alcohol consumption, insufficient intake of fruits and vegetables, and physical inactivity. Moreover, occupational exposure to carcinogens is also highly relevant in oncology. According to the WHO, these carcinogens include physical agents (ionizing and non-ionizing radiation), biological agents (e.g., hepatitis B and C viruses, HIV), and a wide range of chemical agents identified by the IARC. Addressing these factors is essential, especially given that 30% to 50% of cancer cases are considered preventable^10^. 

In this context, the present study aims to conduct a systematic scoping review to evaluate the applications, advantages, and limitations of GIS in cancer surveillance with occupational relevance. It seeks to highlight the critical role of GIS in understanding cancer and its multifactorial determinants, emphasizing the need for robust epidemiological surveillance systems to monitor occupational carcinogen exposure. Additionally, GIS facilitate adjustments in healthcare service delivery to better meet demand, improve cancer care planning by prioritizing quality attributes such as accessibility, timeliness, and relevance, and support the formulation of informed public policies. 

## Materials and Methods

The present study was conducted following the methodological guidelines of the Joanna Briggs Institute (JBI)^10^,^11^ and the PRISMA-ScR protocol for scoping reviews^12^. A literature search was conducted across four main databases: PubMed, Embase, Scopus, and Bireme (BVS), using selected keywords and tailored search strategies to identify relevant articles. Key terms were adapted to the thesauri of each database, employing specific algorithms to optimize search sensitivity. The review was reported in compliance with the PRISMA statement for scoping reviews, and the protocol was registered in the INPLASY platform under code 202430058^13^. The article search covered the period from 2018 to 2022, was limited to publications in English, Portuguese, and Spanish, and employed a broad, sensitive strategy to identify the most relevant literature, considering specific keywords (Table 1). The final searches were consolidated and managed for screening using the Rayyan.ai web application^14^. 

**Table 1:** Details of the Search Strategy and Sources

Databases	Search Details
PubMed	"Neoplasms"[Mesh] AND (("Public Health"[Mesh]) AND ("Public Health Surveillance"[Mesh] OR "Public Health Informatics"[Mesh] OR "Public Health Practice"[Mesh] OR "Public Health Administration"[Mesh] OR "Environment and Public Health"[Mesh] OR "Public Health Systems Research"[Mesh] )) AND "Geographic Information Systems"[Mesh]
EMBASE	('public health surveillance'/exp OR 'public health surveillance' OR (('public'/exp OR public) AND ('health'/exp OR health) AND ('surveillance'/exp OR surveillance))) AND ('geographic information system' OR 'geographic mapping' OR 'geographic distribution') AND (2018:py OR 2019:py OR 2020:py OR 2021:py OR 2022:py) AND ('neoplasm' OR 'malignant neoplasm' OR cancer)
Scopus	(TITLE-ABS-KEY ( neoplasms ) OR TITLE-ABS-KEY ( cancer ) AND TITLE-ABS-KEY ( "geographic information systems" ) ) AND PUBYEAR > 2017 AND PUBYEAR > 2017
Bireme BVS	(cancer) AND ("geographic information systems") AND (year_cluster: [2018 TO 2022])

 The selection process followed the PCC framework, focusing on adults with cancer (Population), use of Geographic Information Systems (GIS) for epidemiological surveillance of occupational cancer (Context), and GIS as tools for spatial analysis of health variables (Concept). Eligible designs included observational studies (case-control, cohort, cross-sectional, ecological, and case series), experimental studies in humans, and systematic reviews. Non-cancer studies, studies without GIS, and narrative reviews were excluded.

 Three pairs of reviewers screened the titles and abstracts of the studies, retrieving full texts for those meeting the selection criteria. Disagreements were resolved by consensus with a fourth reviewer. To minimize potential biases in study inclusion, several strategies were implemented. First, titles and abstracts were screened independently by three pairs of reviewers to reduce selection bias. Second, predefined inclusion and exclusion criteria were strictly applied and discussed in advance to ensure consistency in decision-making. Third, a fourth reviewer resolved any discrepancies by consensus, further reducing subjective influence.

 Extracted data included authors, year, country, cancer type, study design, and GIS use (methodology, software, and results). GIS applications were classified into five categories: thematic mapping, spatial modeling, web GIS, GIS/GPS tools, and spatiotemporal clustering. Finally, a fourth reviewer verified the data, which were stored in Mendeley Data^15^. A narrative synthesis was conducted, complemented by descriptive statistics and frequency measures for selected impact indicators.

**Figure 1:**
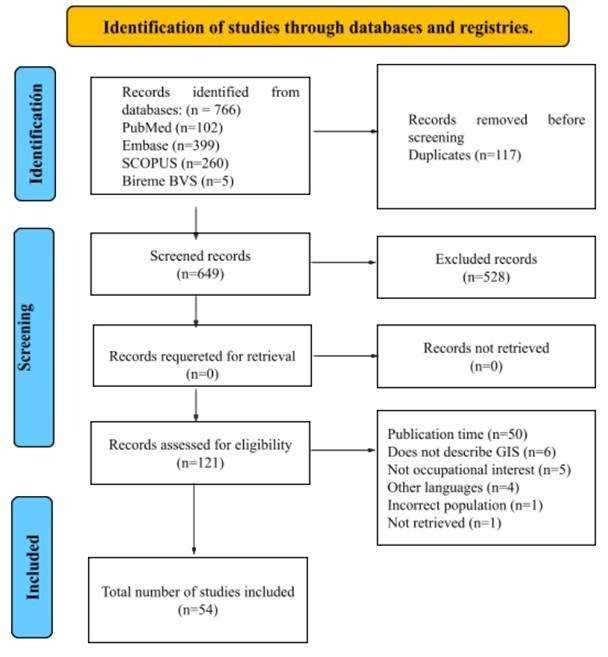
PRISMA flow diagram summarizing the study selection process

 In accordance with the JBI scoping review methodology recommendations^10^,^11^, a qualitative analysis of the identified variables was performed, complemented by simple descriptive statistics. Although some studies reported quantifiable data, the high heterogeneity of study designs, populations, indicators, and reporting formats limited the feasibility of conducting a valid quantitative synthesis. Instead, the extracted data were organized and analyzed thematically to identify patterns in GIS applications related to occupational cancer, allowing for a comprehensive understanding beyond numerical aggregation. Database searches identified 766 articles; after removing duplicates, 649 studies were screened by title and abstract. After applying the eligibility criteria, 528 articles were excluded. Subsequently, full-text review resulted in 54 studies included in the qualitative synthesis (Figure 1).

## Results

The majority of the selected studies originated from the Americas (n=20), followed by Asia (n=15), Europe (n=10), Africa (n=5), and Oceania (n=3) (see Supplementary Material). Regarding the type of cancer, four studies addressed cancer in general, with breast cancer being the most studied (n=14), and blood and non-myeloid lymphatic system cancers the least studied (n=1). The predominant study designs included ecological studies (n=31), cross-sectional studies (n=6), and case-control studies (n=6), with cohort studies being the least reported (n=1). Most studies analyzed data at the county, district, or provincial level, or from population registries. Over the observed period, the year with the most publications was 2018 (25.45%), followed by 2019 and 2021 (23.63% each), 2022 (16.33%), and 2020 (10.9%). 

The most investigated types of cancer were breast, lung, gastroesophageal, and colorectal cancer, with a smaller proportion of studies focusing on liver, skin, melanoma, ovarian, and prostate cancer. Additionally, 20% of the articles focused on occupational cancers, such as lung cancer and malignant mesothelioma. Afurther 16.36% explored the association between industrial emissions, such as dioxins, PM2.5 particles, cadmium, and pesticides, and various cancer types. Regarding the GIS software used, ArcGIS was the most frequently cited, with 33 references (60.00%), followed by QGIS with 6 references (10.91%). Another 6 articles (10.91%) did not specify the GIS employed. The remaining proportion was distributed across other GIS, and 9.09% of the articles (5 references) employed more than one GIS in their analyses (Table 2). 

**Table 2:** Geographic Information Systems documented in the review

SIG	n (%)	Author
ArcGIS	33 (60.00%)	Sahar L, et.al^3^* Coudon T, et. al^16^** Danjou AMN, et. al^17^ Jiang A, et. al^18^ O'Callaghan-GordoC, et. al^19^** VoPham T, et.al^20^ Ahmadi A, et. al^21^ Salmeron B et.al^22^ Khoshdel A, et. al^23^ Jiang F, et.al^24^ Motlana MK, et. al^25^ Ekenga, C.C. et. al^26^ Lysaniuk B, et. al^27^ Moustafa M, et. al^28^ Bux RK, et. al^29^ Virgilsen LF, et. al^30^ Elbasheer MMA, et. al^31^ Gurney J, et. al^32^ Zhou K, et.al^33^ Krówczyńska M, et. al^34^ Slavik CE, et. al^35^ Rubenstein J H, et. al^36^ Wang N, et. al^37^ Kiani B, et. al^38^ Solikhah S, et. al^39^ Khan JR, et. al^40^ Shafiq J, et. al^41^ Flytkjær Virgilsen L, et. al^42^ Kennedy, C, et. al^43^*** Amadou A, et.al^44^ Stangl S, et. al^45^ Krówczyńska M, et. al^46^ VoPham T, et. al^47^
QGIS	6 (10.91%)	Bunyatisai W, et. al^48^ Rankantha A, et. al^49^ Raoof M, et.al^50^ Chan J, et. al^51^*** Carles C, et. al^52^ Yee EK, et. al^53^
ArcMap	4 (7.27%)	Knapp GC, et. al^54^ Won YJ, et.al^55^ VoPham T, et.al^56^ Zhai Y, et. al^57^
GeoDa	2 (3.64%)	Sullivan, M.Wet.al^58^ Ma K, et.al^59^
Geocoding API	2 (3.64%)	Tailor TD, et.al^60^ Stephens JM, et.al^61^
SaTScan	1 (1.82%)	Jaber SM, et. al^62^
SIG Geocuba	1 (1.82%)	Cuéllar-Luna, et. al^63^
SIG Not specified	6 (10.91%)	Wang Y, et. al^64^ Tanaka H, et. al^65^ Jackson L et. al^66^ Omidakhsh N, et. al^67^ Dilekli N, et. al^68^ Soffian SSS,et. al^69^

*Additionally, SaTScan was used; **Additionally, ArcMap was used; ***Additionally, GeoDa was used.

**Spatial analysis method used**

Regarding the spatial analysis method, it was documented that 30.91% of the articles (17 studies) used descriptive methods, 50.91% used analytical methods (28 studies), and 12.72% employed mixed methods. The remaining proportion corresponded to articles that, due to their study design, did not directly apply any form of spatial analysis (Table 3). 

The reviewed studies reported a variety of relevant oncology indicators, with 43.63% (n=24) addressing morbidity and mortality indicators, particularly the incidence and geographic distribution of types of cancer such as head and neck, lung, mesothelioma, gastrointestinal, breast, prostate, ovarian, and brain tumors. Additionally, 12.5% of these studies reviewed gender distribution, and 25% focused on mortality. Access indicators, present in 23.6% of the articles (n=13), evaluated travel time and distance to oncology centers. one study showed that greater distance to a radiotherapy center was associated with increased mortality (R2=0.70; GWR R2=0.74). Moreover, access-relted studies evaluated the relationship between longer distances and lower treatment adherence in 23% of cases, as well as diagnosis at advanced stages in 15.3% of cases. Environmental indicators (23.6%, n=13) examined pollutants such as dioxins and heavy metals. Significant findings include increased cancer risk linked to candium exposure in several locations (SRR=3.27) and an association between glyphosate expossure and thyroid cancer (OR=1.33). Inequity indicators, present in 1.81% of the studies, revealed the relationship between environmental toxicity and poverty (RR=5.34) and the limitations for ovarian cancer staging in rural areas, associated with lower survival (HR=2.05). Regarding timeliness indicators, one study found that greater distances to oncology services increased diagnostic intervals by approximately 6 days (β=0.09, p<0.001). Finally, one article introduced an induced demand indicator, where access to breast cancer screening showed significant spatial autocorrelation (Moran’s I=0.803) and variation in response to invitations based on demographic factors and distance (p< 0.001) (Table 4). 

**Table 3:** Spatial analysis methods and mapping types documented in the reviewed articles

Method	Spatial Analysis or Mapping Type	Reference(s) n
Descriptive	Point pattern analysis, dot density maps, and hotspots^33^,^48^,^54^ Choropleth maps^23^,^34^,^39^,^41^,^55^,^60^,^61^ Centroid analysis^61^* Buffer vector analysis^27^,^28^ Probabilistic method, kriging, and spatial interpolation^31^,^37^*,^39^ Isopleth maps / Isoline maps^37^*,^45^ Map overlay^20^	3 7 1 2 3 2 1
Analytical	Clusters,^18^,^21^,^22^,^26^,^36^,^50^,^58^*,^59^,^62^,^66^ Linear regression, logistic regression, generalized regression, Pearson's method, Poisson regression (Besag-York-Mollié (BYM))^17^,^19^,^20^,^29^,^30^,^32^,^42^,^44^,^47^,^49^,^52^,^56^,^57^,^58^*,^65^,^67^ Spatial prediction methods and natural breaks^35^,^64^	10 16 2
Mixed	Atmospheric dispersion model^16^ Combination of one or more descriptive and analytical methods^18^,^25^,^34^,^40^,^43^,^46^,^51^,^53^	1 8

*Includes more than one descriptive or analytical method. Classification based on: Valbuena-Garcia y Rodríguez-Villamizar^70^

**Table 4:** Categories of indicators identified according to the type of cancer studied

Type of cancer studied / Type of Indicator	Morbidity/Mortality	Access	Modeling of environmental agents	Coverage	Inequality	Timeliness	Induced demand
Breast	n=6^21^,^22^,^25^, ^31^,^57^,^63^	n=5^28^,^42^,^45^,^51^,^54^	n=4^16^,^17^,^19^,^44^	n=0	n=0	n=0	n=1^40^
Lung, malignant mesothelioma	n=6^27^,^34^,^37^,^49^,^63^,^64^	n=3^30^,^51^,^60^	n=2^24^,^46^	n=0	n=0	n=0	n=0
Gastrointestinal includes esophagus, stomach, colon, pancreas, liver, gallbladder, and biliary tract.	n=8^23^,^24^,^33^,^36^,^38^,^47^,^55^,^59^	n=6^32^,^33^,^42^,^51^,^53^,^65^	n=3^18^,^20^,^47^	n=150	n=0	n=0	n=0
Head and neck includes thyroid, larynx, oropharynx, and brain tumors.	n=5^27^,^48^,^52^,^55^,^64^	n=0	n=1^67^	n=0	n=0	n=0	n=0
Testicle, ovary, and prostate	n=3^27^,^55^,^63^	n=2^42^,^51^	n=0	n=0	n=1^58^	n=0	n=0
Skin, melanoma, Kaposi's sarcoma	n=1^25^	n=1^30^	n=1^43^	n=0	n=0	n=0	n=0
Leukemia	n=0	n=1^65^	n=0	n=0	n=0	n=0	n=0
General (unspecified) cancer	n=3^39^,^62^,^66^	n=3^41^,^42^,^61^	n=3^26^,^29^,^35^	n=0	n=0	n=1^42^	n=0

## Discussion

The studies reviewed highlight the significant advancements and contributions of Geographic Information Systems (GIS) in data collection, integration, and analysis, particularly from diverse sources and large datasets. GIS have proven instrumental in mapping cancer cases and identifying associations between the disease and various factors^71^. However, GIS application is not without challenges. Key limitations include issues related to data accuracy and quality, difficulties in extrapolating findings, and complexities in modeling environmental agents. Additionally, the use of GIS in health studies is hindered by geolocation inaccuracies due to incomplete or imprecise data, such as reliance on postal codes, and the omission of critical factors like traffic or transportation dynamics^72^. Many analyses overlook key social and economic variables, limiting the scope for causal interpretation. Technical challenges in environmental monitoring—such as scarce historical data and inconsistent methods— further hinder analyses. Additionally, outdated or incomplete records weaken model reliability. Nonetheless, GIS remain powerful tools with great potential to enhance cancer surveillance and guide targeted public health actions^73^. 

Occupational cancer is a significant public health problem worldwide. Studies conducted in the 1990s, such as the Carex project, led by the Finnish Institute of Occupational Health, identified that 23% of the working population across 15 European countries was exposed to carcinogenic agents, which corresponds to approximately 32 million workers^74^. Occupational exposure to carcinogenic substances has been linked to various types of cancer, with estimates suggesting that 30% to 50% of such cases could be prevented^75^. In this context, GIS have facilitated the identification of risk areas and their association with social determinants of health. GIS play a pivotal role in identifying cancer incidence and mortality patterns, high-risk regions, and disparities in healthcare access. Moreover, they support the creation of health intervention programs, enabling the development of prevention plans and occupational health policies^76^. 

The review revealed a growing global interest in the spatial epidemiology of cancer, particularly in high-burden countries such as the United States and China. Consistent with global incidence patterns, most studies focused on breast and lung cancers and predominantly employed ecological designs, a common approach in geospatial health research due to its feasibility for population-level analysis. Notably, several studies addressed cancers related to occupational and environmental exposures, including lung cancer and malignant mesothelioma associated with asbestos, and gastrointestinal cancers linked to dioxin and cadmium exposure^16^. These findings are consistent with previous reports emphasizing the usefulness of GIS in environmental health surveillance and in identifying localized risk factors^20^,^34^. One study conducted in China exemplified this application by using GIS tools to map cadmium-contaminated areas and demonstrate their association with increased gastrointestinal cancer risk, reinforcing the value of geospatial analysis in guiding targeted public health interventions^77^. 

Regarding head and neck cancers, occupational asbestos exposure emerged as a significant factor, extending its relevance beyond the traditional focus on lung cancer and mesothelioma. Other studies explored the disruption of circadian rhythms in night shift workers and its association with hepatocellular carcinoma, a relationship affecting 10%-30% of night workers globally. The review also highlighted a study documenting the link between thyroid cancer and pesticide exposure, which is particularly relevant given the prominence of the agricultural industry and the direct chemical risks faced by workers in this sector^78^. Furthermore, studies on ultraviolet radiation and electromagnetic fields underscored the environmental and occupational risks associated with skin cancer and brain tumors^79^. 

Indicators of morbidity, mortality, and environmental agent modeling were among the most frequently analyzed variables, reflecting the focus on their public health implications. In contrast, indicators of healthcare access and inequity were less commonly addressed, suggesting the need for further research in these areas. GIS facilitate the analysis of healthcare access and inequities, as demonstrated by a study linking exposure to contaminated environments with unfavorable socioeconomic conditions^26^,^80^. Moreover, a gap in the use of coverage and timeliness indicators was noted, highlighting the potential for future studies to optimize healthcare resources and enhance the coverage of cancer prevention and treatment services^81^. 

GIS remain valuable tools for identifying spatial cancer risks and guiding interventions, even in studies that present limitations, such as variability in data quality, lack of application standardization, and restricted generalizability of findings^82^. Integrating GIS with emerging technologies, such as artificial intelligence and machine learning, holds promise for predictive modeling and strengthening occupational cancer surveillance^83^. It is recommended that future research incorporate quantitative analyses, such as meta-analyses, whenever data quality and homogeneity allow. 

Finally, the application of analytical and mixed methodologies in the reviewed studies underscores the potential of GIS to integrate environmental, social, and economic analyses into a comprehensive approach to cancer research. However, persistent challenges include geocoding accuracy and the extrapolation of individual-level data, especially in rural areas. Additionally, there is a need to account for multiple health determinants and individual exposure levels. 

## Conclusions

Geographic information systems (GIS) are crucial tools for cancer surveillance, providing valuable insights into exposure patterns and the social/environmental determinants. Their application supports the formulation of public policies, healthcare planning, and preventive strategies, especially in occupational health. GIS' integration of key indicators highlights its potential to transform cancer research. Future research should focus on integrating GIS with emerging technologies such as artificial inteligence and machine learning to enhance predictive modeling and risk assessment. Additionally, incorporating new indicators, like genetic factors, and promoting interdisciplinary collaboration will further advance the field, improving prevention strategies and cancer control, and enhancing the quality of life for workers.

## Supplemental Material

**Table 5:** Characteristics of the studies included in the review

No	Qualification of the article	Author	Year of publication	Country of publication	Type of study	Aim (s)
1	Spatial Distribution of Head and Neck Cancer in Chiang Mai, Thailand^48^	Bunyatisai W, Chakrabandhu S, Sripan P, Rankantha A, Prasitwattanaseree S, Chitapanarux I.	2022	Thailand	Ecological	Determine the geographic patterns in the incidence of head and neck cancer in Chiang Mai province with emphasis in oropharynx cancer given the specific risk factors.
2	Geospatial access predicts cancer stage at presentation and outcomes for patients with breast cancer in southwest Nigeria: A population-based study^54^	Knapp GC, Tansley G, Olasehinde O, Wuraola F, Adisa A, Arowolo O, Olawole MO, Romanoff AM, Quan ML, Bouchard-Fortier A, Alatise OI, Kingham TP	2021	Nigeria	Ecological	Analyze the relationship between geospatial access stage of the breast cancer and survival at a tertiary-referral center in southwestern Nigeria.
3	Spatial and temporal characteristics of cancer in the period from 2004 to 2013 in the Hashemite Kingdom of Jordan^62^	Jaber SM; Ibbini JH; Hijjawi NS; Thnaibat JJ; Nimri OF	2018	Jordan	Ecological	Study spatial and temporal characteristics of cancer in 12 governorates of Jordan for the period 2004-2013, for identifying cancer risk factors and developing control plans in that country.
4	GIScience and cancer: State of art and trends for cancer surveillance and epidemiology^3^.	Sahar L, Foster SL, Sherman RL, Henry KA, Goldberg DW, Stinchcomb DG, Bauer JE.	2019	USA	Review	Make maps to reveal the patterns of the disease in relation to local environmental factors with the hope of shedding light on disease etiology
5	Risk patterns of lung cancer mortality in northern Thailand^49^	Rankantha A, Chitapanarux I, Pongnikorn D, Prasitwattanaseree S, Bunyatisai W, Sripan P, Traisathit P.	2018	Thailand	Ecological	Identify the risk patterns for lung cancer mortality in 81 districts of the northern region of Thailand.
6	Geographical Variations and Trends in Major Cancer Incidents throughout Korea during 1999-201355^55^	Won YJ, Jung KW, Oh CM, Park EH, Kong HJ, Lee DH, Lee KH	2018	Korea	Ecological	Describe the temporal trends and district-level geographica
7	Systematic failure to operate on colorectal cancer liver metastases in California^50^	Raoof M, Jutric Z, Haye S, Ituarte PHG, Zhao B, Singh G, Melstrom L, Warner SG, Clary B, Fong Y.	2020	USA	Ecological	Characterize the variation in liver resection utilization rates in California from the population perspective as treatment increases survival.
8	A general method for evaluating the effects of air pollutants on lung cancer prevalence^64^	Wang T, Yue S, Zheng B, Hao Z, Chen J.	2018	China	Modeling mathematics	Quantitatively assess the effects of air pollutants and their relation with lung cancer prevalence in all the studied districts in Tianjin, China.
9	Access to radiotherapy and its association with cancer outcomes in a high-income country: Addressing the inequity in Canada^51^	Chan J, Polo A, Zubizarreta E, Bourque JM, Hanna TP, Gaudet M, Dennis K, Brundage M, Slotman B, Abdel-Wahab M.	2019	USA	Ecological	Associate cancer outcomes with the level of access to radiotherapy across Canada
10	Development and performance evaluation of a GIS-based metric to assess exposure to airborne pollutant emissions from industrial sources^16^	Coudon T, Danjou AMN, Faure E, Praud D, Severi G, Mancini FR, Salizzoni P, Fervers B.	2019	France	Cohort study (validation study of an exposure assessment method)	Develop and assess performances of an exposure metric based on a GIS through comparison with a validated dispersion model to estimate historical industrial dioxin exposure for its use in a case-control study nested within a prospective cohort.
11	Long-term airborne dioxin exposure and breast cancer risk in a case-control study nested within the French E3N prospective cohort^17^	Danjou AMN, Coudon T, Praud D, Lévêque E, Faure E, Salizzoni P, Le Romancer M, Severi G, Mancini FR, Leffondré K, Dossus L, Fervers B.	2019	France	Case-control study	Estimate breast cancer risk associated with exposure to airborne dioxins using GIS methods and historical exposure data.
12	Cancer Mortality and Long-Term Environmental Exposure of Cadmium in Contaminated Community Based on a Third Retrospective Cause of Death Investigation of Residents Living in the Guangdong Province from 2004 to 2005^18^	Jiang A, Gong L, Ding H, Wang M.	2021	China	Ecological	Compare the temporal and geographic trends of cancer in China with a focus on the long-term exposure to soil cadmium (Cd) pollution.
13	Residential proximity to green spaces and breast cancer risk: The multicase-control study in Spain (MCC-Spain)^19^	O'Callaghan-Gordo C, Kogevinas M, Cirach M, Castaño-Vinyals G, Aragonés N, Delfrade J, Fernández-Villa T, Amiano P, Dierssen-Sotos T, Tardon A, Capelo R, Peiró-Perez R, Moreno V, Roca-Barceló A, Perez-Gomez B, Vidan J, Molina AJ, Oribe M, Gràcia-Lavedan E, Espinosa A, Valentin A, Pollán M, Nieuwenhuijsen MJ.	2018	Spain	Case-control study	Investigate the associations between presence of urban green areas, presence of agricultural areas, and surrounding greenness and breast cancer risk, and to assess whether these associations are mediated by physical activity and levels of air pollution.
14	Geographic Access to Cancer Treatment in Japan: Results from a Combined Dataset of the Patient Survey and the Survey of Medical Institutions in 2011^65^	Tanaka H, Ishikawa KB, Katanoda K.	2018	Japan	Cross‑sectional	Describe the distribution of travel time for hospital admissions of patients with cancer and identify underlying factors.
15	Ambient PM2.5 air pollution exposure and hepatocellular carcinoma incidence in the United States^20^	VoPham T, Bertrand KA, Tamimi RM, Laden F, Hart JE.	2018	USA	Ecological	Prospectively examine the association between particulate matter air pollution < 2.5 µm in diameter (PM2.5) exposure and hepatocellular carcinoma in the United States.
16	Incidence pattern and spatial analysis of breast cancer in Iranian women: Geographical information system applications^21^	Ahmadi A, Ramazani R, Rezagholi T, Yavari P	2018	Iran	Ecological	Perform a spatial analysis and determine the incidence pattern of breast cancer in the Islamic Republic of Iran.
17	Emissions of dioxins and dioxin-like compounds and incidence of hepatocellular carcinoma in the United States^47^	VoPham T, Bertrand KA, Fisher JA, Ward MH, Laden F, Jones RR.	2022	USA	Ecological	Examine the association between ambient dioxin air emissions from industrial sources and the risk of Hepatocellular carcinoma in United States
18	Assessing health disparities in breast cancer incidence burden in Tennessee: geospatial analysis^22^	Salmeron B, Mamudu L, Liu X, Whiteside M, Williams F.	2021	USA	Cross‑sectional	Explore the geographic disparities patterns in breast cancer incidence in Tennessee by Appalachian and non-Appalachian County of residence.
19	Spatio-temporal analysis of colorectal cancer using to geographic information system in the Iranian military community during the period 2007-2016^23^	Khoshdel A, Alimohammadi M, Sepandi M, Alimohamadi Y, Jalali P, Janani M.	2020	Iran	Retrospective ecological study	Conduct a temporal trend analysis of incidence rate, and also to identify regional spatial clusters of colorectal cancer in the Iranian military community using spatio-temporal analysis for the period 2007–2016.
20	Missing information in statewide and national cancer databases: Correlation with health risk factors, geographic disparities, and outcomes^58^	Sullivan MW, Camacho FT, Mills AM, Modesitt SC.	2019	USA	Cross‑sectional	Assess ovarian cancer patients at multiple levels (institutional, state, and national) and to analyze differences in outcomes and patient characteristics based on grade.
21	Spatial distribution and clusters of pancreatic cancer mortality in Shandong Province, China^24^	Jiang F, Chu J, Chen X, Zhang J, Fu Z, Sun J, Lu Z, Guo X, Xu A	2019	China	Ecological	Explore the geographic distribution and risk clusters of pancreatic cancer mortality between 2011 and 2013 in Shandong, China, and detect the differences between urban and rural areas
22	Spatial Distribution of Cancer Cases Seen in Three Major Public Hospitals in KwaZulu- Native, South Africa^25^	Motlana MK, Ginindza TG, Mitku AA, Jafta N	2021	KwaZulu-Natal, South Africa	Ecological	Describe cancer incidence and spatial distribution of cancer cases seen at 3 main public oncology facilities in KwaZulu-Natal.
23	Cancer in an historical Washington DC African American population and its geospatial distribution^66^	Jackson L, Jackson H, Mohammed M, Guthrie N, Kim S, Okolo R, Jackson F.	2018	USA	Ecological	Assess the frequencies of the types of cancer present among Cobb Collection individuals, compare these data with current research on cancer in the African Americans, and assess the pattern of cancer expression, including its geospatial distributions, as a cause of death between 1931 and 1969 in an historic African American subgroup and compare this pattern with the historic and contemporary patterns of cancer etiology and incidence
24	Cancer risk from air toxics in relation to neighborhood isolation and sociodemographic characteristics: A spatial analysis of the St. Louis metropolitan area, USA^26^	Ekenga CC, Yeung CY, Oka M	2019	USA	Ecological	Investigate the spatial distribution of carcinogenic air toxics in the St. Louis metropolitan area and identify if sociodemographic characteristics are associated with exposure to carcinogenic air toxics.
25	Stratification of the emergence risk of non communicable diseases associated with the environmental contamination in Cuba^63^	Cuéllar LL, Maldonado CG, Suárez TS, del Puerto RA, Romero PM	2021	Cuba	Ecological	Identify the variation of the mortality due to lung, breast and prostate cancer and their possible association with the environmental contamination.
26	Using CHALK to Estimate Population at Risk Because of Residence Proximity to Asbestos Processing Facilities in Colombia^27^	Lysaniuk B, Cely-García MF, Giraldo M, Larrahondo JM, Serrano-Calderón LM, Guerrero-Bernal JC, Briceno-Ayala L, Cruz Rodriguez E, Ramos-Bonilla JP.	2021	Colombia	Ecological	Estimate the number of people from the general population living in distance bands from asbestos processing facilities at which an elevated risk of asbestos-related diseases has been reported
27	Surveying and mapping breast cancer services in Ghana: a cross-sectional pilot study in the Eastern Region^28^	Moustafa M, Mali ME, Lopez-Verdugo F, Sanyang O, Nellermoe J, Price RR, Manortey S, Biritwum-Nyarko A, Ofei I, Sorensen J, Goldsmith A, Brownson KE, Kumah A, Sutherland E.	2021	Ghana	Cross-sectional	Define the available services for breast cancer care at hospitals in the Eastern Region of Ghana and identify areas of the region with limited access to care through geospatial mapping
28	Natural and anthropogenic origin of metallic contamination and health risk assessment: TO hydro- Geochemical study of Sehwan Sharif, Pakistan^29^	Bux RK, Haider SI, Batool M, Solangi AR, Memon SQ, Shah ZU, Moradi O, Vasseghian Y.	2022	Pakistan	Ecological	Assessing carcinogenic and non-carcinogenic human health risk due to exposure of metal (loid)s from groundwater consumption.
29	Travel distance to cancer-diagnostic facilities and tumour stage^42^	Flytkjær Virgilsen L, Møller H, Vedsted P.	2019	Denmark	Cohort study	Study the association between different types of cancer with the distance from the patient’s home to the hospital of diagnosis.
30	Spatiotemporal Distribution and Evolution of Digestive Tract cancer cases in Lujiang County, China since 2012^59^	Ma K, Lin Y, Zhang X, Fang F, Zhang Y, Li J, Yao Y, Ge L, Tan H, Wang F.	2022	China	Ecological	Analyze the spatiotemporal distribution and evolution of digestive tract cancer in Lujiang County by using the GIS technology
31	Thyroid Cancer and Pesticide Use in to Central California Agricultural Area: TO Case Control Study^67^	Omidakhsh N, Heck JE, Cockburn M, Ling C, Hershman JM, Harari A.	2022	USA	Case-control study	Examine environmental factors (pesticides) that influence the risk of thyroid cancer
32	Improved Geocoding of Cancer Registry Addresses in Urban and Rural Oklahoma^68^	Dilekli N, Janitz A, Campbell J.	2020	USA	Ecological	Geocoding addresses in rural areas with poorer quality of information to formulate hypotheses related to the distribution of cancer in Oklahoma.
33	Spatial distribution of breast cancer in Sudan 2010-2016^31^	Elbasheer MMA, Alkhidir AGA, Mohammed SMA, Abbas AAH, Mohamed AO, Bereir IM, Abdalazeez HR, Noma M.	2019	Sudan	Cross‑sectional	Estimate the prevalence of breast cancer and determine its spatial distribution country-wide.
34	Equity of travel required to access first definitive surgery for liver or stomach cancer in New Zealand^32^	Gurney J, Whitehead J, Kerrison C, Stanley J, Sarfati D, Koea J.	2022	New Zealand	Cross‑sectional	Examine the distance and the time taken to access to surgical care and compare these factors between Maori and European patients with liver or stomach cancer
35	Geographic hotspot detection for late-stage hepatocellular carcinoma: a novel approach to cancer control^33^	Zhou K, Thompson LK, Liu L, Terrault NA, Cockburn MG.	2022	USA	Cross‑sectional	Describe a population-based geospatial approach to identifying areas with high late-stage hepatocellular carcinoma burden for intervention.
36	Spatial analysis of asbestos exposure and occupational health care in Poland during the period 2004-2013^46^	Krówczyńska M, Wilk E.	2018	Poland	Ecological	Gather data on asbestos exposure in Poland, developing a PostgreSQL database to implement geoinformation techniques for reducing diseases developed due to asbestos exposure.
37	Industry and geographic patterns of use and emission of carcinogens in Ontario, Canada, 2011-2015^35^	Slavik CE, Kalenge S, Demers PA.	2018	Canada	Ecological	Assess Ontario Toxics Reduction Act (TRA) and Canada National Pollutant Release Inventory (NPRI) ability of monitor trends in the use and the emission of carcinogens by industry in Ontario
38	Circadian misalignment and hepatocellular carcinoma incidence in the United States^56^	VoPham T, Weaver MD, Vetter C, Hart JE, Tamimi RM, Laden F, Bertrand KA.	2018	USA	Ecological	Examine the association between distance from time zone meridian, a proxy for circadian misalignment, and hepatocellular carcinoma (HCC) risk in the United States adjusting for known HCC risk factors.
39	Clustering of esophageal cancer among white men in the United States^36^	Rubenstein JH, Morgenstern H, Longstreth K.	2019	USA	Ecological	Examine geographic clustering of esophageal cancer in the United States and assess whether that clustering is explained by the distribution of known risk factors for esophageal cancer.
40	Lung Cancer Mortality in China: Spatial and Temporary Trends Among Subpopulations^37^	Wang N, Mengersen K, Tong S, Kimlin M, Zhou M, Wang L, Hu W	2019	China	Ecological	Identify changing spatial and temporal trends of lung cancer mortality rates among subpopulations in China (according to region, age, and sex).
41	Residential proximity to power lines and risk of brain tumor in the general population^52^	Carles C, Esquirol Y, Turuban M, Piel C, Migault L, Pouchieu C, Bouvier G, Fabbro-Peray P, Lebailly P, Baldi I.	2020	France	Case-control study	Investigate the association between residential proximity to power lines and brain tumors among adults in France by using a geographical information system.
42	Association between heavy metals and colon cancer: an ecological study based on geographical information systems in North-Eastern Iran^38^	Kiani B, Hashemi Amin F, Bagheri N, Bergquist R, Mohammadi AA, Yousefi M, Faraji H, Roshandel G, Beirami S, Rahimzadeh H, Hoseini B.	2021	Iran	Ecological	Explore the spatial pattern of age-standardized incidence rate of colon cancer and its potential association with the exposure level of the amount of heavy metals existing in rice produced in north-eastern Iran.
43	Geographic Characteristics of Various Cancers in Yogyakarta Province, Indonesia: A Spatial Analysis at the Community Level^39^	Solikhah S, Perwitasari DA, Rejeki DSS	2022	Indonesia	Ecological	Determine the spatial distribution of cancer cases in Yogyakarta Province
44	Area-level determinants in colorectal cancer spatial clustering studies: A systematic review^69^	Soffian SSS, Nawi AM, Hod R, Chan HK, Hassan MRA.	2021	Malaysia	Systematic review	Identify and synthesize available evidence on clustering patterns of cancer colorectal incidence, specially related to the associated determinants
45	Geographic impact on access to care and survive for non- curative esophagogastric cancer: to population-based study^53^	Yee EK, Coburn NG, Zuk V, Davis LE, Mahar AL, Liu Y, Gupta V, Darling G, Hallet J.	2021	Canada	Ecological	Investigate the association between distance from cancer facilities and rates of medical oncology consultation, receipt of cancer-directed therapy, and survival.
46	Residential area and screening venue location Features associated with spatial variation in breast cancer screening invitation response rates: An observational study in Greater Sydney, Australia^40^	Khan JR, Carroll SJ, Warner-Smith M, Roder D, Daniel M.	2021	Australia	Cross‑sectional	Assess small-area variation in BCS invitation response rates (IRRs) and associations between small-area BCS IRR, sociodemographic factors, BCS venue distance and venue location features in Greater Sydney, Australia.
47	Radiotherapy service needed in the Pacific Island countries^41^	Shafiq J, Gabriel GS, Barton MB.	2020	Australia	Modeling mathematics by density population	Provide a quantitative estimation of the effect of establishing new radiotherapy (RT) facilities on patient access through GIS modelling of population density and service availability to assess the best location for a new RT center when there are multiple competing locations.
48	Cancer diagnostic delays and travel distance to health services: A nationwide cohort study in Denmark^42^	Flytkjær Virgilsen, L.; Møller, H.; Vedsted, P.;	2019	Denmark	Cohort study	Investigate the association between distance to health services and the intervals the cancer diagnostic pathway, and explore whether the diagnostic difficulty of the cancer influences this association.
49	Developing indices to identify hotspots of skin cancer vulnerability among the Non- Hispanic White population in the United States^43^	Kennedy C, Liu Y, Meng X, Strosnider H, Waller LA, Zhou Y.	2021	USA	Ecological	Explore spatial clusters to identify vulnerable groups to skin cancer.
50	Geographic Access to CT for Lung Cancer Screening: A Census Tract-Level Analysis of Cigarette Smoking in the United States and Driving Distance to a CT Facility^60^	Tailor TD, Choudhury KR, Tong BC, Christensen JD, Sosa JA, Rubin GD.	2019	USA	Ecological	Determine, at a census tract level, the geographic distribution of US smokers and their driving distance to an ACR-accredited CT facility.
51	Disparities in accessibility to evidence-based breast cancer care facilities by rural and urban areas in Bavaria, Germany^45^	Stangl, S.; Rauch, S.; Rauh, J.; Meyer, M.	2021	Germany	Ecological	Identify areas with access restricted to its installation of careful of breast cancer closest
52	Chronic long-term exposure to cadmium air pollution and breast cancer risk in the French E3N cohort^44^	Amadou A, Praud D, Coudon T, Danjou AMN, Faure E, Leffondré K, Le Romancer M, Severi G, Salizzoni P, Mancini FR, Fervers B.	2020	France	Cohort study	Estimate the risk of breast cancer associated with long-term exposure to airborne cadmium pollution, and its effect according to molecular subtype of breast cancer (estrogen receptor negative/positive [ER−/ER+] and progesterone receptor negative/positive [PR−/PR+])
53	Travel burden associated with granulocyte colonystimulating factor administration in a Medicare Aged population: a geospatial analysis^61^	Stephens JM, Bensink M, Bowers C, Hollenbeak CS.	2018	USA	Ecological	Examine the travel burden related to prophylactic granulocyte colony-stimulating factors G-CSF injections after chemotherapy in the US.
54	The impact of left truncation of environmental exposure case–control studies: evidence from breast cancer risk associated with airborne dioxin^57^	Zhai Y, Amadou A, Mercier C, Praud D, Faure E, Iwaz J, Severi G, Mancini FR, Coudon T, Fervers B, Roy P.	2022	France	Case-control study	Analyze the bias induced by left truncation in estimating breast cancer risk associated with exposure to airborne dioxins. Simulations were run with exposure estimates from a GIS-based metric
